# Remote Patient Monitoring at Home in Patients With COVID-19: Narrative Review

**DOI:** 10.2196/44580

**Published:** 2024-11-19

**Authors:** Justien Cornelis, Wendy Christiaens, Christophe de Meester, Patriek Mistiaen

**Affiliations:** 1 Belgian Health Care Knowledge Centre Brussels Belgium

**Keywords:** COVID-19, coronavirus disease, telemonitoring, remote patient monitoring, review, pandemic, at-home monitoring, implementation, health care, patient care

## Abstract

**Background:**

During the pandemic, health care providers implemented remote patient monitoring (RPM) for patients experiencing COVID-19. RPM is an interaction between health care professionals and patients who are in different locations, in which certain patient functioning parameters are assessed and followed up for a certain duration of time. The implementation of RPM in these patients aimed to reduce the strain on hospitals and primary care.

**Objective:**

With this literature review, we aim to describe the characteristics of RPM interventions, report on patients with COVID-19 receiving RPM, and provide an overview of outcome variables such as length of stay (LOS), hospital readmission, and mortality.

**Methods:**

A combination of different searches in several database types (traditional databases, trial registers, daily [Google] searches, and daily PubMed alerts) was run daily from March 2020 to December 2021. A search update for randomized controlled trials (RCTs) was performed in April 2022.

**Results:**

The initial search yielded more than 4448 articles (not including daily searches). After deduplication and assessment for eligibility, 241 articles were retained describing 164 telemonitoring studies from 160 centers. None of the 164 studies covering 248,431 patients reported on the presence of a randomized control group. Studies described a “prehosp” group (96 studies) with patients who had a suspected or confirmed COVID-19 diagnosis and who were not hospitalized but closely monitored at home or a “posthosp” group (32 studies) with patients who were monitored at home after hospitalization for COVID-19. Moreover, 34 studies described both groups, and in 2 studies, the description was unclear. In the prehosp and posthosp groups, there were large variations in the number of emergency department (ED) visits (0%-36% and 0%-16%, respectively) and no convincing evidence that RPM leads to less or more ED visits or hospital readmissions (0%-30% and 0%-22%, respectively). Mortality was generally low, and there was weak to no evidence that RPM is associated with lower mortality. Moreover, there was no evidence that RPM shortens previous LOS. A literature update identified 3 small-scale RCTs, which could not demonstrate statistically significant differences in these outcomes. Most papers claimed savings; however, the scientific base for these claims was doubtful. The overall patient experiences with RPM were positive, as patients felt more reassured, although many patients declined RPM for several reasons (eg, technological embarrassment, digital literacy).

**Conclusions:**

Based on these results, there is no convincing evidence that RPM in COVID-19 patients avoids ED visits or hospital readmissions and shortens LOS or reduces mortality. On the other hand, there is no evidence that RPM has adverse outcomes. Further research should focus on developing, implementing, and evaluating an RPM framework.

## Introduction

The COVID-19 pandemic caused health care services around the globe to rapidly respond to the needs of people diagnosed with SARS-CoV-2 infection [1]. However, health care services in most countries were underprepared for this large-scale biological event and were stretched [2]. At the beginning of the pandemic, it was especially difficult to increase hospital capacity and upscale staffing levels. During the summer of 2020 with the possibility of a second wave in mind, health care providers started to adjust their preparedness and response protocols in order to be better prepared. During the subsequent waves of the pandemic, characterized by increased infection rates, the development and expansion of new health care services were boosted.

On the one hand, there was a need for community management of people who were infected and were presenting with symptoms, especially to reduce the strain on hospital resources (intensive care bed capacity, staffing, ventilators, etc) and health care worker exposure (personal protective equipment, etc). On the other hand, there was a need to increase responsiveness as primary care was overwhelmed and emergency departments (EDs) noticed that patients were receiving the care they needed too late. Remote patient monitoring (RPM) involves an interaction between health care professionals and patients who are at different locations, during which certain patient parameters are assessed and followed up for a certain duration of time. The idea arose to remotely monitor patients at home as much as possible in order to prevent these patients from going to the general practitioner (GP) and avoid hospitalization. GPs initiated their own RPM by means of telephone calls and remote assessments of parameters (eg, heart rate, blood pressure, oxygen saturation, weight, symptoms) measured by patients or their relatives at home or by ambulatory care nurses (data were transferred via electronic devices [Bluetooth, digital modes, broadband, wireless, etc]). Moreover, hospitals with prior experience in RPM for chronic pathologies, in which RPM was effective [3-5], started to develop care paths to spare hospital beds. This remote interaction involves several elements, such as patients, RPM staff, interaction content, and equipment. These elements and therefore the characteristics of RPM might differ owing to the simultaneous development of RPM across health care settings and health care providers around the globe, the quick initiation endorsed by the crisis situation, and the varying available resources and experiences.

COVID-19 was an unfamiliar pathology characterized by a rapidly changing nature and context. Owing to the novelty of the pathology and the variations in clinical presentations across infection waves and in formats of remote care, health care professionals indicated that valid risk stratification scales and assessment tools were lacking. Patients with COVID-19 (suspected) infection who had deteriorating symptoms, which usually occurred within 14 days after illness onset, needed to be identified in time [6]. A decision-aid report published on June 1, 2021 [7] mentioned intensified home care involving telemonitoring performed at least 2 to 3 times a day, with assessment of clinical parameters measured by patients, caregivers, or health care professionals. Based on the information obtained, advice could be given and therapy could be initiated (thromboprophylaxis, oxygen therapy, corticosteroids, other drugs [paracetamol and antibiotics], etc). Moreover, short-term oxygen therapy could be initiated at home. Patients eligible for remote monitoring could be sent home with RPM and oxygen therapy instead of being hospitalized. However, in this specific pathology, there was limited evidence on the most successful health care model for community management of COVID-19 patients and RPM.

Telemonitoring can be used to recognize and treat changes in the patient’s health status as a stand-alone approach (eg, early detection) or as part of a telerehabilitation intervention. Moreover, the adoption of new care models is often challenged by unfamiliarity with program eligibility, services, and logistics, leading most providers to select the care option with which they are familiar (ie, traditional hospitalization and ED or GP visits). Patients can be reluctant to try out new approaches of care. For COVID-19, patients raised many questions, were very anxious, and requested admission for specialized care.

Health care organizations and professionals mainly initiated RPM in 2 specific groups of patients with COVID-19: (1) “prehosp” group with patients who had a suspected or confirmed COVID-19 diagnosis and were admitted to the GP’s practice or ED but were not hospitalized and instead closely monitored at home, and (2) “posthosp” group with patients who were monitored at home after hospitalization for COVID-19. This study will focus on both groups and differentiate between the groups regarding outcome measures.

The purpose of this study is to find out if noninvasive RPM has been applied for COVID-19 patients to avoid hospital admission (prehosp) and to discharge patients earlier from the hospital (posthosp) (ie, number of hospital readmissions). Moreover, in the prehosp and posthosp groups, the study aims to investigate whether RPM in patients with COVID-19 is feasible or has an effect on the following outcomes: length of stay (LOS), number of ED visits, mortality, costs, savings, and patient experiences.

## Methods

Several types of databases and sources were consulted as many COVID-19 studies were not yet published in traditional databases (Table 1). A combination of different searches in several database types (traditional databases, trial registers, daily Google searches, and daily PubMed alerts) was performed from March 2020 to December 2021. The searches were updated on April 16, 2022, but selection for relevant articles was limited to randomized controlled trials (RCTs).

**Table 1:** Database type and source.

Database type	Source	Retrieved articles, n
Traditional database	PubMed, CINAHL, EMBASE, LISSA, and Cochrane Library	2520
Specifically developed for COVID-19 literature	NBCI [8], BVSalud [9], Cochrane Library [10], EBSCO Medical [11], COVID-19 Reviews [12], CEBM [13], and CADTH [14]	1910
Preprint server	bioRxiv [15], arXiv [16], Archives Ouvertes France [17], JMIR Preprints [18], and medRxiv [19]	0
Clinical Trials register	ClinicalTrials.gov [20], Clinical Trials Register Europe [21], WHO Clinical Trials Registry Platform [22], and Clinical Trials Database Belgium [19]	18
Worldwide web	Google Advanced and Google Scholar by means of the Publish or Perish interface	—^a^

^a^Not applicable as daily and monthly searches were performed continuously throughout the analysis of the retrieved articles in order not to miss newly published studies.

**Figure 1:**
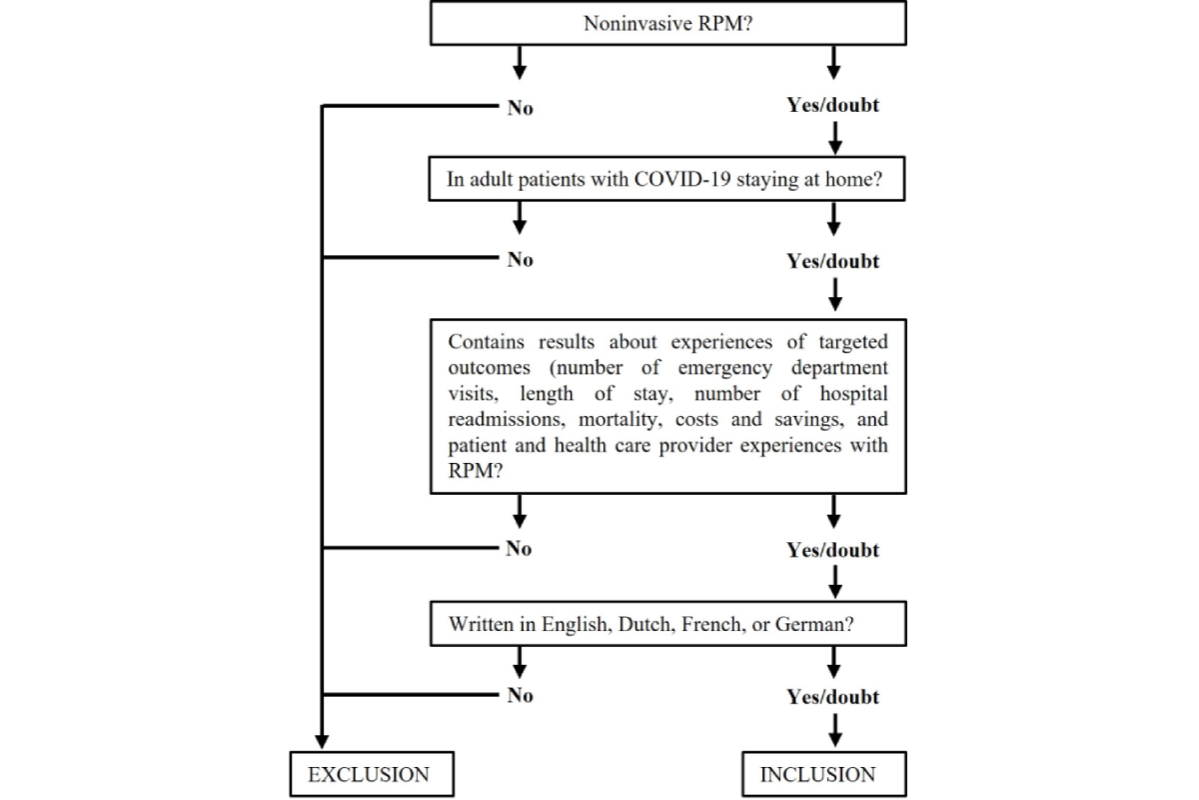
Inclusion and exclusion process. RPM: remote patient monitoring.

The adapted PICO(T) search was used. The search strategy aimed to include patients with COVID-19 residing at home (P), who were receiving noninvasive RPM (I), to follow their clinical status. Keywords were combined to describe the patient population ((Covid-19 OR Covid* OR corona OR Sars-Cov2) AND (home OR discharge OR post-hospital)) and intervention ((Telemonitor OR “remote monitor” OR “remote patient monitoring” OR “remote home monitoring” OR “hospital at home” OR “virtual visit” OR “virtual round” OR “virtual hospital” OR telehealth OR telemedicine OR smartphone OR wearable OR “mobile health” OR mhealth)). In some databases, specially developed search filters for COVID-19 were used. No specific keywords were added to the search, but selection criteria were set a priori. Articles were selected based on main outcomes (O) if the citations reported on the experiences of patients, ED visits, hospital readmission, LOS, or mortality, or if the costs or savings of telemonitoring were reported. In the initial search, all study types were included (T) irrespective of the comparator (C). The updated search was limited to RCTs. Articles were excluded if they concerned invasive RPM, involved patients residing at locations other than home, were not describing one of the main outcomes, or were published in a language other than English, French, Dutch, or German (Figure 1). Reference lists were checked for any topic-related studies. Expert opinions and recommendations on ongoing unpublished studies or other relevant data were gathered. The corresponding authors of studies were contacted to obtain any missing information or data. If means or SDs were not mentioned, these values were obtained by recalculation.

The references were retrieved and imported into EndNote for deduplication. Initially, 1 researcher (PM) screened the results from the electronic searches to select relevant citations based on titles and abstracts. Full-text articles were retrieved and evaluated based on the set selection criteria (Figure 1). In case of uncertainty, a second investigator (JC) evaluated the citation and consensus was sought during a meeting.

Owing to the crisis situation, we adopted an “ongoing” search (over more than 1 year) in order to detect articles published across the COVID-19 waves of infections. Therefore, we could not provide an overall estimation of hits. In addition, this approach of continuously searching may have provided us with more articles at a faster pace compared with systematic searching methods in traditional databases.

The data from the studies were extracted by 1 researcher (PM). The primary data extracted were related to the main outcomes (ie, number of ED visits, number of hospital readmissions, LOS, mortality, costs and savings, and patient experiences). The secondary data extracted were related to the general characteristics of the studies (ie, authors, publication year, study design, origin/country, and specific team/center), the characteristics of the patient population (eg, prehosp or posthosp, number of patients included, confirmed presence of COVID-19 infection, stage of COVID-19, place of residence, severity of symptoms, presence of comorbidities, risk profile, selection), and the characteristics of the intervention (eg, intervention elements, platform used, health care practitioners involved in monitoring, parameters monitored, duration of monitoring, number of clinical interventions, number of alerts). Data on costs, savings, and patient experiences were retrieved. In case the outcomes for a combined group of prehosp and posthosp were reported, the articles were left out from the main outcome analysis, except for the patient satisfaction outcome.

## Results

### Overview

The initial search yielded more than 4448 articles (Table 1). In addition, periodical searches in traditional databases and daily searches were performed. As these did not provide the total hits per search, they have not been included in this number. After deduplication and assessment for eligibility, 241 articles were retained, which described 164 telemonitoring studies from 160 centers [1,6,23-261].

### General Characteristics of the Studies

Studies were conducted across the globe in over 28 countries. Most studies were from the United States (n=64), United Kingdom (n=15), Australia (n=11), Spain (n=11), and Italy (n=10). Studies were also conducted in Argentina, Belgium, Bolivia, Brazil, Canada, China, Czech Republic, Egypt, France, Gambia, Germany, India, Iran, Ireland, Israel, Japan, Malaysia, Peru, Portugal, Saudi Arabia, South Korea, Switzerland, and The Netherlands.

All the included articles concerned observational studies, with some of them including a kind of comparison arm (eg, patients who received telemonitoring in certain areas versus patients from another area who presumably had not received telemonitoring, patients with symptoms versus asymptomatic patients, patients with RPM at home versus patients with RPM in a quarantine hotel, patients receiving low-intensity RPM versus patients receiving high-intensity RPM, or prehosp RPM versus posthosp RPM). Seven studies [67,81,118,133,165,211,253] applied a matched control study design in which RPM was compared to no RPM and patient characteristics (such as comorbidities and risk profiles) were taken into account in a weighted way. Among these matched control studies, 6 [67,118,134,203,211,253] concerned prehosp patients, while 3 [81,118,165] described posthosp patients. With a search update, 3 small RCTs were included. Since limited RCTs were retrieved, no methodological assessment was performed.

### General Characteristics of the Patient Population

The research population size varied from 10 to 43,103. Fifty studies were small-scale studies with less than 100 patients, but there were also 6 studies [47,123,124,147,167,253] with more than 10,000 patients. Overall, the included 164 studies covered 248,431 patients.

The patients included in the retrieved studies had proven or suspected COVID-19 infection and were residing at home. The moment of RPM initiation varied among patients and included the day of being suspected with COVID-19, the day of the first symptoms, the day of a positive test, the day of an ED visit, the day of worsening of symptoms, and the day of hospital discharge.

Among the studies, 96 concerned only prehosp patients and 32 concerned only posthosp patients. Moreover, 34 studies described both groups, and in 2 studies, it was unclear if prehosp or posthosp patients were considered.

The patients were in a certain stage of the COVID-19 disease (asymptomatic, immediately after suspicion of COVID-19 infection, mild symptomatic, or severe disease presentation) and had comorbidities or risk factors (which were not always described in the studies). Some of the studies (eg, [39,100,136,163]) included only high-risk patients (eg, aged ≥65 years and 1 comorbidity), while others (eg, [28,79,146,160]) included only low-risk patients or did not select the population based on risk stratification. The way in which and the criteria on which the risk was assessed differed or was not described. In some cases, deterioration risk assessment was used to select patients, while in others, it was used to adapt the intervention to the risk profile (increasing frequency of measuring parameters, additional parameters to follow, adapted alert settings, etc). Some studies focused on special populations with COVID-19, such as oncological patients [59,62,63,70,84,109,122,133-135,137,140,153,179,225], children [64,218,238], liver transplant patients [239], and pregnant or postpartum women [170,180,223,240,245].

### General Characteristics of the Intervention

Across the studies, a variety of health care professionals, such as nurses, nurse practitioners, physician assistants, physiotherapists, respiratory specialists, psychologists, social workers, dieticians, medical and nursing students, GPs, and medical specialists, remotely monitored patients with COVID-19. Sometimes a stepped approach was applied (eg, nurses performed the monitoring, but in case of deterioration, the monitoring was transferred to a medical specialist).

In many studies, existing RPM staff and infrastructure for managing telemonitoring in patients with other diseases were used and extended. RPM studies were mainly hospital initiated, but in some cases [1,26,68,79,131,138,162,176,203,219,252], primary care professionals also applied a form of RPM. The sizes of the telemonitoring teams described in the studies varied from a single professional to a larger multidisciplinary telemonitoring team. Sometimes a specialized telemonitoring team, external of a hospital, was used (eg, [102,174]). The articles rarely mentioned full-time equivalents. Many studies used volunteers, and retired and redeployed health care professionals (eg, [26,47,49,57,68,80,82,126, 128,166,206,212,232]). In addition, administrative and technical staff members were added. The staff members assigned to conduct RPM differed across studies and settings and were not always clearly described.

Patients needed a smartphone, computer, or tablet for information exchange and a number of measuring devices (eg, thermometer, saturation meter, blood pressure meter, pulse meter), either as a separate device for each parameter or a single device for a combination of parameters (eg, smartwatch and in-ear device). Some measuring devices took measurements automatically, sometimes in a continuous way, and were sometimes connected via the internet or Bluetooth to the patient’s electronic device. In addition to objective registration of physiologic measurable parameters, studies also used daily surveys monitoring subjective variables such as dyspnea, fatigue, and pain. All parameter data were sent to and processed on an information and communication system to provide health care professionals with numerical and graphical insights into patient functioning. This information and communication system could either be stand-alone or integrated into the electronic patient record of a hospital or a GP. The devices and digital infrastructure used to conduct RPM differed across studies and settings and were not always clearly described.

The interaction mode between the patient and the RPM team could involve 1-way communication (patient to RPM team) or 2-way communication. Different combinations of telephone audio calls, video calls, text messages, and specially created software platforms were used. At the time of initiating the telemonitoring, a combination was sometimes made with a home visit (by one or more health care professionals; eg, [102,233,262]) for instructions or technology set-up. The interaction mode for conducting RPM differed across studies and settings and was not always clearly described.

Owing to the lack of controlled studies, a large variation was found in patient functioning variables that were monitored across the different studies (eg, general well-being, fatigue, coughing, diarrhea, smell, mobility, temperature, heart rate, respiratory rate, shortness of breath, oxygen saturation). The parameter that was most often monitored was oxygen saturation, followed by subjective dyspnea. The ways in which these variables were assessed varied (patient self-assessment, assessment by a health care professional [at site or remotely], or assessment by means of a connected device). Moreover, alerting cutoffs for each parameter varied. In some cases, the numbers of monitored variables (and devices) were scaled up or down depending on patients’ conditions. The assessment frequency varied widely from once per 2 days and 5 times per day to 24×7 continuously (eg, [48,53,72,89,90,92,121,131,142,154,163,164,172,188, 196,201,209,210,250]) for some parameters, and the frequency could vary during the course depending on the presenting symptoms [174]. In both the prehosp and posthosp groups, telemonitoring was sometimes accompanied (eg, [102,134,152,177,178,185,197,203,205,208,211,221,233,238,256,260]) by other interventions, such as oxygen therapy, antibiotics, antipyretics, anticoagulants, corticosteroids, hydroxychloroquine, and lopinavir or ritonavir. However, details about dose, frequency, and duration were mostly lacking. Many articles did not mention whether there were co-interventions. From the articles, it was not clear to what extent these co-interventions influenced the measured functioning variables and outcomes.

Based on the data received (the interaction content), reactions from the RPM team were provided. These reactions varied and included (1) no reaction as long as parameters were within the set limits (“no news is good news” strategy), (2) a reassuring reaction toward the patient each time parameters were uploaded to tell them they were received and normal, (3) an automatically generated signal or a call to patients to reassess a parameter when this was suspicious, (4) a call to a GP or registered nurse that a parameter was suspicious and further investigation or a home visit could be useful, (5) a call to the patient to visit the GP or ED for further check-up, and (6) a call to the patient to immediately present to the hospital for admission. Sometimes deviating parameters were first discussed within the RPM team and with specialist consultants before a reaction. It was unclear what types of interventions were deployed on what types of alerts for which parameters and if all these reactions were systematically registered in the systems by the RPM team. The interaction content and reactions of the telemonitoring team differed across studies and settings and were not always clearly described.

Overall, a high heterogeneity in the technology used and the characteristics of the interventions (ie, amount of staffing, devices and digital infrastructure, details on the health care settings, details on the health care system, interaction mode, interaction content, and reactions of the telemonitoring team) was observed among studies. Moreover, the lack of RCTs limited the control of monitored variables and the risk stratification of patients. The described results were not provided by all studies as the information was not always available. Therefore, it was not possible to compare RPM between different COVID-19 interventions and between studies.

### Results for the Main Outcomes

There were large variations in outcomes that were measured and reported. Studies discussed process outcomes (number of patients who refused RPM, eg, [40,81,119,147,153,171,177,191,199,251]; number of alerts, eg, [23,29,50,58,59,62,66,81,92,98,111,117,124,127, 135,142,154,155,165,166,174-176,181,185,204,210,250]; number of interactions and reactions of the RPM team, eg, [70,72,81,86,90,117,152,154,174,179,203,212,225,238,240,245,252,254,256]; number of technical problems, eg, [50,91,105,175,244]; and duration of RPM), clinical outcomes (number of ED visits, number of hospital readmissions, and mortality), economical outcomes (cost of RPM, hospital days avoided, and cost savings, eg, [6,28,35,43,61,75,76,85,96,114,126,130,139,144,158,159, 172,173,176,178,185,187,202-204,241,251,256,261]), and experiences of health care professionals (eg, [28,37,38,49,51,53,70,75,87,92,110,131,139,149, 160,170,172,173,181,187,189,200,230,236]) and patients (eg, [25,28-30,34,36-38,47,48,53,54,57,62,63,65,68, 70,72,75,83,86,87,91,92,96,99,100,107,109, 114,116,119-121,124,126,130,131,135,139,144,149,151, 153,158,163,165,168,169,172-174,178,181,187,189,199, 207,208,213,214,220,222,224,226,235,236,243,247,248,254]). The periods wherein the outcome measurements were performed and registered were rarely clear. The lack of RCTs limited controlling for these outcomes.

Some studies (eg, [150,174]) presented separate analyses for low- and high-risk patients with substantive differences between them. In these studies, low-risk patients received lower intensity interventions. The outcomes presented in studies that did not make this risk differentiation should be read cautiously.

As noted in the description of the outcomes, there was a large variation in reported outcomes. Moreover, the lack of RCTs limited controlling for these outcomes. Furthermore, among studies that reported on similar outcomes, the measurement procedure or time of measurement (eg, in which period was ED admission or mortality measured, when was it decided to stop measuring) mostly differed from study to study, making the pooling of results very difficult. However, we extracted these data from the studies as much as possible and succeeded in pooling the results. With regard to clinical outcomes, for both groups, we retrieved the duration of RPM, the number of ED visits, hospital readmission, and mortality. For the hospitalized patients in the posthosp group, LOS was retrieved. As seen in Table 2, more studies reported on these outcome measures in the prehosp group. The duration of RPM varied from a single day to several weeks. The median duration of RPM was 10 and 13.6 days for the prehosp and posthosp groups, respectively. ED visits were more frequent in the prehosp group (11.2%) than in the posthosp group (6%). Similar percentages of hospital admissions (6.4%) in the prehosp group and readmissions (5.4%) in the posthosp group were noted. In both the prehosp and posthosp groups, low mortality rates (0.15% and 0%, respectively) were reported. The median LOS for hospitalized (posthosp) patients was 6 days (with a large variation between 1.7 and 38 days). It was not possible to correlate these outcomes to certain variables, such as the RPM duration to certain patient population risk profiles, comorbidities, or end points, for the reasons mentioned earlier. Concerning specific patient populations, RPM appears to be feasible in these patients. More detailed information can be found in another publication [263].

**Table 2:** Description (pooling results) of studies reporting on the targeted clinical outcomes.

Outcome variable	Prehosp group				Posthosp group		
	Studies, n	Median (range)	Percentile (P25-P75)	Studies, n		Median (range)	Percentile (P25-P75)
Duration of RPM^a^ (days)	33	10.0 (3.5-21.8)	8.0-13.1	16		13.6 (3.1-90.0)	11.8-20.5
Emergency department visits (%)	54	11.2 (0.0-36.0)	5.7-19.9	13		6.0 (0.0-15.8)	2.8-10.3
Hospital readmissions (%)	81	6.4 (0.0-30.4)	3.1-11.4	23		5.4 (0.0-22.2)	2.0-10.5
Mortality (%)	55	0.15 (0.0-8.8)	0.0-1.1	14		0.0 (0.0-4.2)	0.0-1.4
Length of stay (days)	—^b^	—	—	11		6.0 (1.7-38.0)	4.0-10.0

^a^RPM: remote patient monitoring.

^b^Not applicable.

We described the clinical outcomes reported in the studies applying a matched control design separately. An overview of the prehosp group is provided in Table 3, and an overview of the posthosp group is provided in Table 4.

**Table 3:** Clinical outcomes in the prehosp group in matched control studies.

Study	Participants, n		ED^a^ visits at 30 days				ED visits at 90 days				30-day hospital admission				Mortality at 30 days				Mortality at 60 days		
	I^b^	C^c^	I, n (%)	C, n (%)	Adj OR^d^ (95% CI)	I, n (%)		C, n (%)	Adj OR (95% CI)	I, n (%)		C, n (%)	Adj OR (95% CI)	I, n (%)		C, n (%)	Adj OR (95% CI)	I, n (%)		C, n (%)	Adj OR (95% CI)
Beaney et al [253]	639	14,982	192 (30.1)	3568^e^ (23.8)	1.37 (1.16-1.63)	—^f^		—	—	152 (23.8)		3180^e^ (21.2)	1.59 (1.32-1.91)	9 (1.4)		430^e^ (2.9)	0.48 (0.25-0.93)	—		—	—
Delgado et al [211]	3488	4377	489 (14.0)	252 (5.7)	0.06 (0.04-0.07)	—		—	—	211 (6.1)		141 (3.2)	1.93 (1.56-2.41)	3 (0.1)		12 (0.3)	0.32 (0.12-0.72)	5 (0.1)		16 (0.4)	0.34 (0.16-0.67)
Dirikgil et al [67]	55	110	—	—	—	—		—	—	5 (9.1)		30 (27.0)	0.27 (0.10-0.73)	—		—	—	—		—	—
Misra-Hebert et al^g^ [118]	2672	1950	273 (10.2)	193 (9.9)	1.03 (0.76-1.39)	382 (14.3)		275 (14.1)	1.01 (0.78-1.31)	302 (11.3)		242 (12.4)	0.90 (0.68-1.20)	—		—	—	—		—	—
Pritchett et al^h^ [134]	71	116	7 (9.9)	18 (15.5)	0.59 (0.24-1.51)	—		—	—	3 (4.2)		15 (12.9)	0.33 (0.09-1.17)	0 (0.0)		4 (3.4)	0.17 (0.01-3.30)	—		—	—

^a^ED: emergency department.

^b^I: intervention group, group receiving remote patient monitoring.

^c^C: control group.

^d^Adj OR: adjusted odds ratio.

^e^Recalculated from OR.

^f^Not applicable.

^g^Based on the number mentioned for outpatients who did not present first to the ED.

^h^Concerning cancer patients with COVID-19.

**Table 4:** Clinical outcomes in the posthosp group in matched control studies.

Study	Participants, n			ED^a^ visits at 30 days				30-day hospital admission				Mortality at 14 days				Length of stay (days)		
	I^b^	C^c^	I, n (%)		C, n (%)	Adj OR^d^ (95% CI)	I, n (%)		C, n (%)	Adj OR (95% CI)	I, n (%)		C, n (%)	Adj OR (95% CI)	I^e^		C^e^	Adj OR (95% CI)
Gordon et al [81]	225	1061	11 (4.9)		46 (4.3)	NS^f^	3 (1.3)		60 (5.7)	0.22 (0.07-0.71)	—^g^		—	—	5 (3-8)		5 (3-8)	NS
Ye et al [165]	217	192	18 (8.3)		27 (14.1)	NS	15 (6.9)		16 (8.3)	NS	3 (1.4)		4 (2.1)	0.66 (0.15-2.99)	5 (3.9)		4.2 (3.2)	NS

^a^ED: emergency department.

^b^I: intervention group, group receiving remote patient monitoring.

^c^C: control group.

^d^Adj OR: adjusted odds ratio.

^e^Data are presented as mean (range) or mean (SD).

^f^Not significant.

^g^Not applicable.

Regarding the clinical outcomes in the prehosp group, 4 studies [118,134,211,253] reported on the number of ED visits. Two of them [211,253] found a significantly higher number of ED visits within 30 days for RPM, while the other 2 did not find a difference. Five studies [67,118,134,211,253] reported on the 30-day hospitalization rate. Two studies [211,253] found a significantly higher number of hospital admissions in the RPM group, 2 [118,134] found no significant difference, and 1 [67] found a significantly lower number of hospital admissions in the RPM group. However, the latter study was a small-scale study, and the 30 patients who were “admitted” in the control group consisted of 25 patients who stayed for less than 24 hours. It could be questioned if this should be regarded as a real hospital admission. When these were taken out of the analysis, there was no longer a significant difference. Three studies [134,211,253] reported on the 30-day mortality rate. Two studies [211,253] found significantly less mortality in the RPM group, and 1 study found the same effect at day 60. The third study [134] was a small-scale study in cancer patients with COVID-19, and there was no significant difference.

One study [118] only included patients who presented first in primary care and excluded patients who presented first to the ED. Moreover, another study [134] concerned patients with cancer and COVID-19. Regarding the effect found in 1 article [253], 2 other publications on the same project used another research design investigating a period without the availability of RPM versus a period with the availability of RPM [257] and investigating regions with higher uptake of RPM versus regions with lower uptake of RPM [258]. The pre-post analysis [257] also found a slightly higher ED attendance in the RPM period, but the regional analysis [258] did not show an effect on ED visits and hospital admissions.

Regarding the clinical outcomes in the posthosp group, 2 studies [81,165] from the United States performed a matched control comparison and found no significant difference in ED visits (with more ED visits in the control group). One study [81] found significantly less hospital readmissions with RPM in multivariate analysis. The control group consisted of patients who did not receive RPM (for unclear reasons). Another study [165] found less hospital readmissions with RPM, but the difference was nonsignificant. A nonsignificant difference in the percentage of patients who died within 14 days was found between those referred to RPM and those not referred to RPM [165]. No significant differences were found for LOS.

In summary, there was a large variety in the number of ED visits across prehosp studies and there was no convincing evidence that prehosp or posthosp RPM leads to less or more ED visits. Moreover, there was no convincing evidence that prehosp or posthosp RPM is associated with less or more hospital readmissions. Mortality with prehosp or posthosp RPM was generally low based on the studies presented in Tables 3 and 4, and there was weak to no evidence showing that RPM is associated with lower mortality than non-RPM. Although some of the presented studies reported a lower mortality for patients with RPM, this finding was not significant. No studies reporting on mortality provided statistics on the general mortality rate per country or health care setting, and the studies had a matched control design (no RCTs). There was no evidence that RPM shortens previous hospital LOS.

The initial search (until December 15, 2021) did not identify RCTs. However, searches showed that there are ongoing studies [264-290], of which 9 [264,265,268,276,277,281,286-288] are RCTs in which at-home patients with COVID-19 will be randomized (yes or no RPM). With the database search update (until April 16, 2022) limited to RCTs, 475 hits were screened, and 3 RCTs were retrieved and discussed [276,291,292].

Two small-scale RCTs with 62 [292] and 150 [291] posthosp patients were identified. van Goor et al [292] concluded that remote hospital care for recovering COVID-19 patients is feasible, but there was no increase in hospital-free days in the 30 days following randomization. They found that the mean difference in hospital-free days was 1.7 (26.7 days in the control group vs 28.4 days in the intervention group, 95% CI of difference −0.5 to 4.2; *P*=.11). In the intervention group, the index hospital LOS was 1.6 days shorter (95% CI −2.4 to −0.8; *P*<.001), but the total duration of care under hospital responsibility was 4.1 days longer (95% CI 0.5-7.7; *P*=.03). A per-protocol analysis [291] indicated that patients in the control group were significantly more likely to return to the ED for COVID-19–related reasons than those in the experimental group (7.9% vs 0%; *P*=.03). However, no differences were observed in the intention-to-treat analysis. Satisfaction with outpatient monitoring was rated more highly by the experimental group in both the per-protocol and intention-to-treat analyses. There were no statistically significant differences reported in the health status questionnaire or anxiety scale by the end of follow-up. Thus, both posthosp trials could not demonstrate statistically significant differences in outcomes between the experimental and control groups.

One RCT [276] compared patients without wearable monitoring technology undergoing routine standard of care at the hospital (n=150) to patients diagnosed with COVID-19 undergoing self-quarantine while being closely monitored using a wearable device (n=130) in the prehosp group. Based on the preliminary results, no significant differences in outcomes between the experimental and control groups were seen. The study has not been published yet, and this conclusion is based on the preliminary data available in the clinical trial register.

In summary, no statistically significant differences were observed in the studies, except [292], which showed that the index hospital LOS was shorter for posthosp patients (suggesting an earlier discharge when patients could be followed up at home with RPM after discharge), but the total duration of care under hospital responsibility was significantly longer. The results of the RCTs are in line with the results of the matched control studies.

### Results on Costs and Savings

Several articles [6,28,29,35,36,43,45,48,55,61,67,75,76,81, 83,85,96,97,114-116,118,126,130,134,139,141,144,150, 157-159,165,172,173,176,178,185,187,202-204,214, 241,251,256,261] included information on the costs of the intervention or made claims on savings with RPM in terms of avoided ED visits, avoided hospital admissions, and reductions in LOS (sometimes expressed in monetary values). The details on the costs and claims on savings were assessed. All these claims were in favor of RPM. However, it needs to be considered that none of these claims and conclusions are based on RCTs. Only a few studies used some kind of comparison group, and the findings are mainly based on expert opinion. In most articles claiming savings, a clear methodology was lacking. Therefore, the scientific base for these claims is doubtful.

### Results on Patient Experiences

Overall, 73 articles [25,28-30,34,36-38,47,48,53,54,57, 62,63,65,68,70,72,75,83,86,87,91,92,96,99,100, 107,109,114,116,119-121,124,126,130,131,135,139,144, 149,151,153,158,163,165,168,169,172-174,178,181,187-189, 199,207,208,213,214,220,222,224,226,235,236,243, 247,248,254] mentioned an indicator of patient experience. In general, patient reports were very positive about RPM. Patients mainly experienced a feeling of reassurance.

However, this overall positive picture might be skewed, because several studies only included patients who already had some digital proficiency and were familiar with smartphone use [167]. Moreover, in most studies, patient satisfaction questionnaires were only answered by some patients who received RPM, increasing the chance for self-selection bias. Some studies (eg, [40,81,119,147,153,171,177,191,199,251]) reported that RPM was offered but patients declined it for several reasons (eg, feeling good enough and too much technological embarrassment expected).

## Discussion

### Principal Findings

The objective of this study was to find out if noninvasive RPM has been used among COVID-19 patients to avoid hospital admissions (prehosp) and to discharge patients earlier from the hospital (posthosp) (ie, number of hospital readmissions). Moreover, in the prehosp and posthosp groups, it aimed to investigate whether RPM is feasible and has an effect on the following outcomes in patients with COVID-19: LOS, number of ED visits, mortality, costs and savings, and patient experiences.

None of the 160 original studies (241 articles) covering 248,431 patients reported on the presence of a randomized control group. Among the studies, 96 described a “prehosp” group with patients who had a suspected or confirmed COVID-19 diagnosis and who were not hospitalized but closely monitored at home, 32 described a “posthosp” group with patients who were monitored at home after hospitalization for COVID-19, and 34 described both groups. In 2 studies, the descriptions were unclear.

All studies aimed to lower the pressure on hospital resources or capacity by avoiding ED visits and hospital readmissions and shortening hospital LOS. In the prehosp and posthosp groups, there was a large variation in the number of ED visits (0%-36% and 0%-16%, respectively) and no convincing evidence that RPM leads to less or more ED visits or hospital readmissions (0%-30% and 0%-22%, respectively). Moreover, there was no evidence that RPM shortens LOS. The studies focused on the timely upscaling of health care interventions in case of possible deterioration of the patient, avoiding deterioration and mortality, plausible cost savings, and reassuring patients. Mortality was generally low. Most papers claimed that savings and overall patient experiences with RPM were positive.

### Considerations

With regard to the characteristics of patients with COVID-19, in the prehosp group, some studies focused on high-risk patients, while others focused on low-risk patients (or somewhere in between). Some studies did not provide this information. Focusing on low-risk patients implies that a higher number of patients should be monitored and consequently more devices are needed. Therefore, there is a higher workload for the RPM team. On the other hand, it provides more certainty that patients showing deterioration are detected, which is certainly an advantage in a pandemic involving a disease course that is largely unknown, and that patients with silent hypoxia can be better detected (contributing more to the goal of early detection before escalation). Focusing on high-risk patients limits the number of patients who need RPM and may ensure that patients with the highest risk are monitored and deterioration is detected in a timely manner. The best choice in the case of an unknown disease is probably to follow-up all patients and consider end points to define prognostic variables. However, there was limited time or resources to do this. In the future, RPM could target both groups but with a differentiated approach (such as number of parameters to be followed, frequency of monitoring, type of devices, and stepped RPM team). Regarding posthosp RPM studies, it was remarked [293,294] that most studies did not use clear criteria to decide which patients could be discharged earlier and followed by RPM. Objective discharge criteria were generally lacking or were not reported (such as afebrile, oxygen independency, and no medication needed). With less criteria applied, more patients could leave the hospital and free up beds (some studies reported on a LOS of 1 day). However, when patients had a longer LOS, they also required a higher complexity of needed postdischarge care and probably had a higher chance of deterioration (some studies reported on patients who were admitted during weeks at the intensive care unit). There is limited clarity regarding when posthosp RPM is useful, which are the end points of RPM, and when the change toward teleconsultation [295] or telerehabilitation [296] can best be made.

Moreover, valid risk stratification scales and assessment tools for patients with COVID-19 were lacking. As stated previously, throughout the pandemic, attempts were made to construct valid risk stratification scales. Formulating clear criteria for safe discharge and establishing end points for RPM follow-up after hospital discharge could be useful. Gavin et al [255] showed that the simplified HOSPITAL score is an applicable instrument to triage patients with COVID-19 for hospitalization according to their risk for potentially avoidable readmissions. Moreover, other studies [262,297-300] have provided useful information on the relationship between patient characteristics and risk for readmission after hospital discharge. However, the number of studies examining risk factors for hospital readmission and postdischarge mortality is small, and sometimes their quality is low owing to various reasons [301].

Regarding the effects of RPM on LOS, several articles stated that LOS was shortened because of the implementation of posthosp RPM. However, it was seen that the rate of readmission in posthosp patients differed greatly and that the timeframe (7, 15, 30, and 60 days after discharge) was not always mentioned. A shorter LOS does not mean much if these patients need to be rehospitalized soon after discharge, and there are indeed indications that a shorter LOS is related to higher rates of readmission (ie, “Short-stay hospitalization had significantly increased odds of rehospitalization within 7 days” [262]; “However, patients who were readmitted had significantly shorter initial LOS (median 7 days (range: 2-54) versus 8 days (range: 2-107), *P*<.001)” [300]; “During the COVID-19 pandemic and its outbreaks, the lack of hospital beds, medical facilities, and human resources caused patients to be discharged too early, leading to increased hospital readmissions and possible post-discharge deaths” [301]).

The results show that it is difficult to interpret if an ED visit is regarded as “good” or “wrong.” On one hand, RPM aims to timely detect deterioration in order to stop further deterioration. On the other hand, RPM aims to avoid ED visits and hospital readmission. These aims are somewhat contradictory. On detecting deterioration, it could be appropriate to further assess the patient in the ED or admit the patient to the hospital. In this way, a large percentage of ED visits and hospital admissions could be interpreted as not only “success” but also “failure.” On sensitive detection of deterioration, RPM could lead to more ED visits and hospital admissions compared to the absence of RPM, but this would lead to more pressure on hospitals, which is contrary to the aim of RPM.

Regarding mortality, no statistically significant differences were noted [118,165,211,253]. Therefore, the intervention seems to be feasible as there are no indications for reverse unexpected outcomes. In the absence of RCTs, it was impossible to correctly estimate mortality. The overall mortality statistics during a period within a country were not provided. RPM was also applied in special patient populations experiencing COVID-19 and was shown to be feasible. Moreover, when patient experiences were mentioned in articles, they were in general positive as patients felt more reassured. However, many patients declined RPM for several reasons (eg, technological embarrassment, digital literacy), questioning the accessibility of RPM. Most studies only registered these patient experiences descriptively, and qualitative methods could be applied in future studies to indicate the strengths and limitations of RPM for users.

Most RPM studies aimed at reducing the strain on hospital resources and capacity by trying to avoid ED visits and hospital readmissions, and shortening LOS. As explained, we did not find convincing statistically significant evidence on this. Moreover, augmenting RPM interventions could also increase the strain on hospital resources. Since most RPM studies were hospital led, hospital personnel are needed to staff the RPM teams. It was seen that “successful” studies (ie, including many patients), such as those from France (COVIDOM [57,66,166,167,190,200]), Brazil (UNIMED [123]), United States (Kaiser Permanente Virtual Home Care Program [124,147]), and Spain (Telea [50,111,136]), staffed their RPM teams with volunteers, students, and retired personnel. Moreover, many other RPM studies partially staffed their RPM teams with these profiles. This might have led to a reduction in strain on hospital personnel but is of course only a temporary solution and not an option in the long run. Thus, RPM may save a hospital bed but not necessarily hospital personnel. It could be the case if RPM teams would be staffed by primary care personnel, but this scenario would inevitably lead to increased workload for primary care, which was confronted with already high workload during the COVID-19 pandemic and which was considered in the aims of several studies (ie, to reduce workload on primary care professionals).

The authors of the described economic studies themselves acknowledged that their results were still preliminary and should be used with caution. Because of the low quality of these studies, no concrete conclusions can be drawn, except that if RPM really allows to avoid hospitalization and if the cost of RPM is inferior to hospital costs, savings could be made, at least initially. However, it is necessary to further investigate whether there are more complications in RPM patients than in hospitalized patients, which could lead to higher costs in the long run. More studies are therefore needed and RPM in COVID-19 must, based on our results, currently be considered as an alternative if hospitals are overcrowded rather than a cost-effective strategy.

### Future Directions

Although RPM appears feasible to apply, there are many questions remaining concerning the characteristics of the RPM interventions. Characteristics, such as the amount of staffing, the digital infrastructure, details on the health care settings, details on the health care system, the interaction mode, the interaction content, the reactions of the telemonitoring teams, the characteristics of the patients included, and the technologies used to obtain those aims, were very heterogeneously implemented across studies and health care settings. Standardized data should be collected, and the following elements should be clarified.

First, regarding the use of technology, diverse devices were applied across studies ranging from very basic and cheap thermometers to advanced, expensive, connected, multiparameter measuring devices. It will be essential in the future to investigate which parameters are essential to follow and what range of precision or accuracy is needed for the measuring devices. Several studies did not always use measuring devices but relied on a survey, asking for subjective parameters such as rate of fatigue and dyspnea. Some questionnaires were very exhaustive. For that, the need and utility of subjective measurements should also be investigated further.

Second, it was questioned which parameter cutoffs are “safe” (eg, oxygen saturation of 92%) or should the cutoffs be adjusted for each patient individually (depending on comorbidities or risk profile). Consequently, it was questioned when the RPM team should react and which action is appropriate for which parameter cutoff. Moreover, the optimal frequency of parameter measurement is unclear (ie, Is continuous and automated transfer of parameters needed or are previously agreed time points sufficient?). These elements may have consequences for the workforce needed for RPM (and consequently staffing and resources for a team). Many unnecessary alerts require more workforce to react and can lead to alarm tiredness, while too few alerts require less workforce but may cause adverse patient events.

Third, variations in the type and amount of personnel in RPM teams were observed. It remains unclear which health care professionals are the most appropriate and what levels of qualifications are required. Although most studies were hospital led, it remains unclear if this setup environment is superior to primary care–led RPM. Perhaps an RPM team can be replaced or greatly assisted by a kind of virtual care assistant as researched by García Bermúdez et al [302], and the question remains who needs to take up the medical responsibility in this care model.

Finally, what is the role of governments in RPM policy design, upscaling, solving barriers, reimbursement, technology requirements, and setting up research programs and evaluation frameworks for patients with COVID-19 and those with other conditions? There already exist some inspiring articles [303-307] that could be used as starting points.

### Limitations

There were some limitations. First, at the time of our searches (ie, in the heat of the COVID-19 pandemic), studies were published as soon as possible to quickly inform the rest of the world, often at the detriment of quality. We did not perform a quality assessment, but often information was not reported and studies did not include a control group. The lack of RCTs illustrates the difficulty to build up evidence during a health crisis. The absence of RCTs limited controlling for variables to assess the effectiveness of RPM on outcomes. We encountered large heterogeneity across studies in patient populations, monitored variables, monitoring modes, involved health care professionals, and intervention doses and modes, prohibiting combining studies and making overall conclusions on the effects or effectivity of telemonitoring in patients with COVID-19. Moreover, a large part of this information was not described in the retrieved studies. Furthermore, countries had different health care systems and health care organizations, which had different levels of crisis preparedness. COVID-19 had an impact in terms of speed and volume of the population affected, and the consequences on surge capacity differed. The stages in which the studies were conducted differed, leading to high heterogeneity in the described characteristics and outcomes. We aimed to group the studies in several ways, such as country of origin and patient inclusion criteria (risk profiling). However, there were no validated risk-profiling scales for COVID-19 patients.

Second, although we aimed to describe the effectiveness of RPM, we encountered many limitations owing to a lack of randomization and controlling for confounding factors as explained earlier. To provide input on effectiveness, we conducted daily updates and performed an update of the search strategy to detect published RCTs. Only 3 small RCTs were detected, illustrating the difficulty to conduct solid research during a crisis. To assess the cost-effectiveness of RPM in patients with COVID-19, larger RCTs should be prospectively conducted. We came across several publications in which only the intervention was presented without any outcome data. It might be expected that at a later stage, follow-up publications will arrive from these studies with outcome data.

Third, it is important to understand that COVID-19 presented in infection waves and with different clinical presentations. Depending on the wave, patients appeared well during the first few days of infection and deteriorated later. The included articles did not report on vaccination status, but across the period of the pandemic, the hospital capacity changed because of mass vaccination. It is unclear if vaccination had an effect on the clinical status in patients who received RPM across the study period. It was difficult to carry out RCTs because of this continuously changing situation and because of the lower influx of patients due to mass vaccination later.

Fourth, we only searched for publications in English, Dutch, German, or French. There might have been publications in other languages from other countries as COVID-19 spread across the world. We coincidentally came across relevant publications in other languages, which we did not include. Moreover, we encountered English written publications from non-English speaking countries. It may be assumed that these are only the tip of the iceberg of what was ongoing in those countries.

Finally, although this review included results from over 160 research studies covering about 250,000 patients, it provides only a partial view of the evidence, and no in-depth analysis of the outcome data could be performed. Therefore, our conclusions need to be regarded as partial, preliminary, and mainly descriptive. Updates of this review should be performed to have a more conclusive view of the effectiveness of RPM in patients with COVID-19 in the future.

### Conclusions

Telemonitoring in patients with COVID-19 has been used frequently and across the world. As RPM in COVID-19 was developed as a reaction to the pandemic and not as an anticipation, these studies are characterized by high degrees of heterogeneity in the patient population, intervention content, process characteristics, and outcomes. Moreover, there was a lack of RCTs. There is no statistically significant evidence that RPM in patients with COVID-19 is effective in avoiding ED visits and hospital readmissions, and shortening LOS or reducing mortality, but there is also no indication that RPM has reverse unexpected outcomes. The lack of clear evidence does not mean that COVID-19 RPM was not cost-effective, but instead means that no research was set up in such a way that this could be shown. This review led to a list of questions that need to be answered before the best combination of elements and the most cost-effective combination can be defined. It is essential that solid scientific evidence is gathered to standardize COVID-19 RPM and to create a framework to effectively implement and rapidly scale virtual strategies for providing hospital-like care at home. While more convincing evidence on COVID-19 RPM is required, there is enough expert-based and other disease-related evidence to continue with the current RPM practice. We learned from COVID-19 that there is no way back for telehealth, telemedicine, and RPM. However, RPM should be developed and standardized. In this development process, attention should be given to the accessibility and feasibility of RPM.

## Abbreviations

ED: emergency department
GP: general practitioner
LOS: length of stay
RCT: randomized controlled trial
RPM: remote patient monitoring
